# Serrated Polyposis Syndrome in a Single-Center 10-Year Experience

**DOI:** 10.4274/balkanmedj.2017.0322

**Published:** 2018-01-20

**Authors:** Hyun Young Kim

**Affiliations:** 1 Department of Internal Medicine, Seoul National University Bundang Hospital, Seongnam-si, Korea

**Keywords:** Serrated polyposis syndrome, prevalence, cancer, colonoscopy

## Abstract

**Aims:**

Serrated polyposis syndrome is a disease that is often missed in the clinical setting and is associated with colorectal cancer. We investigated the prevalence of SPS and the association between colorectal or other cancers in a 10-year, retrospective data analysis.

**Methods:**

We reviewed complete colonoscopy data obtained from January 2005 through January 2015 at a health-screening centre. Serrated polyposis syndrome was defined on the basis of the criteria established by the 2010 World Health Organization.

**Results:**

Of a total of 53.842 consecutive subjects who underwent complete colonoscopy, 12 (0.022%) patients had serrated polyposis syndrome. All of these cases were under-recognized by the endoscopist or referring physician. The mean patient age was 58.6 years; 67% of the patients were men and 33% were women. No serrated polyposis syndrome patients had a first-degree relative with serrated polyposis syndrome, and no serrated polyposis syndrome patients had colorectal cancer. Two cases (17%) had extra-colonic cancers (prostate cancer and thyroid cancer). Eight cases (67%) had a family history of cancer (stomach, breast, lung, pancreas, prostate and colorectal cancer).

**Conclusion:**

Serrated polyposis syndrome was a rare condition in a 10-year database, and it was diagnosed late in all cases. Serrated polyposis syndrome may be associated with an increased risk of extra-colonic cancer.

Colorectal cancer (CRC) is the third most common type of malignant disorder in the world. The rapidly rising trend in the incidence and mortality of CRC is surprising in westernised Asia, including China, Japan, Singapore and Korea. The worldwide age-standardized rate of CTC per 100.000 population was 24.7 in Korea and the highest in the world in 2012 (1,2).

Serrated polyposis syndrome (SPS), defined by multiple serrated colon polyps, has an association with personal and familial risks of CRC. Diagnostic criteria for SPS were first described in 2000 and recently redefined in 2010 by the World Health Organization (WHO) (3): (1) ≥5 serrated colon polyps proximal to the sigmoid colon with 2 or more of these being >10 mm; (2) any number of serrated polyps proximal to the sigmoid colon in an individual who has a first-degree relative with SPS; or (3) >20 serrated polyps of any size distributed throughout the colon (not all in the rectum).

The association between SPS and personal and familial CRC risk is well established (4,5), although the genetic nature and natural history of this syndrome remain unknown. The aim of this study was to investigate the prevalence and clinicopathological features of SPS in Korea and its association with CRC and extra-colonic cancer.

## MATERIALS AND METHODS

The study population consisted of all subjects who underwent colonoscopy at a tertiary care medical centre from January 2005 through January 2015. The study group consisted of 53.842 asymptomatic subjects aged 22 to 88 years at average risk for CRC who underwent a complete screening colonoscopy examination. All subjects filled out a questionnaire regarding family history of CRC, exercise, alcohol drinking and smoking. Smoking status was categorized into three groups: never, former and current. The Institutional Review Board approved the study (IRB No. B-1512/328-116).

All colonoscopies were performed by five expert colonoscopists (gastroenterologists >1000 colonoscopies) certified by the Korean Society of Gastrointestinal Endoscopy and other endoscopists (<300 colonoscopies) by using a CF-Q260AI/AL or CF-H260AI/AL system (Olympus, Tokyo, Japan). Bowel preparations consisted of 4 L or 2 L of fluid based on polyethylene glycol or sodium phosphate. Our data excluded cases in which incomplete cecal intubation or unfair bowel preparation had occurred. Our quality indicators of colonoscopy were provided by a previous study (6).

Colonoscopists estimated the size of the polyp by using the open biopsy-forceps method or total removal size of the polyp after colonoscopic polypectomy. Serrated lesions were categorized as hyperplastic polyp, sessile serrated adenoma and traditional serrated adenoma.

## RESULTS

Twelve of 53.842 patients (0.022%) were diagnosed with SPS by the WHO criteria. Table 1 shows the baseline demographic data. The mean age (±SD) at diagnosis of SPS was 58.5±9.5 years, and 8 (67%) of the subjects were men.

Among the 12 subjects, no patient was suspected of this syndrome by the endoscopists or referring physician. All of these SPS cases remained unrecognized until the review was conducted. During the review, it was found that five patients had sufficient serrated polyps detected at the index colonoscopy to enable an adequate diagnosis of SPS by the WHO criteria. All of the SPS patients fulfilled the criterion of “≥5 serrated polyps proximal to the sigmoid colon with two or more of these being >10 mm”.

The average number of colonoscopies per patient was 2.3 (range, 1-3). The average number of years of colonoscopic surveillance per patient was 2.3 (range, 2-4). Two patients with SPS developed extra-colonic cancer: thyroid and prostate cancer. A 76-year-old man was diagnosed with prostate cancer and is currently receiving radiotherapy after prostatectomy. A 65-year-old woman was diagnosed with papillary thyroid cancer and has been recovered after thyroidectomy. No subjects had a first-degree relative with SPS. No subjects had CRC. No subjects had more than 30 serrated polyps, and the average size of the largest polyp was 15 mm (Table 2).

## DISCUSSION

The present retrospective analysis of SPS at a single tertiary institution shows the prevalence of SPS in an average-risk population undergoing health-surveillance screening colonoscopy. We found that 0.022% of patients met the WHO criteria for SPS. Of note, SPS was not recognized by the health check-up centre endoscopist, gastroenterology department practice endoscopist or referring physicians (gastroenterologist). Finally, we are missing all of the SPS patients in the health check-up centre or gastroenterology department practices.

The prevalence of SPS in our study was 0.022% (1:4487), which was lower than that of the most well-known prevalence study on SPS of 0.033% (1:3000) (7) and higher than that of a recent Japanese study (0.014%) (8). Previous SPS prevalence reports were divided by Eastern or Western studies and by screening with colonoscopy or screening with stool occult blood tests. Previous SPS prevalence reports varied widely from 0% to 0.66% in a Western systematic review (9). This systematic review showed that some studies revealed that patients with colonoscopy-based screening programs had a lower SPS prevalence (0-0.09%) (10,11,12) than patients pre-selected for colonoscopy or sigmoidoscopy with a stool test (0.03%-0.66%) (13,14).

In this study, failure to diagnose or missing SPS at screening colonoscopy was a common event among patients with SPS, occurring in 12 (0.022%) SPS patients. However, it is important to recognize that this is a relatively uncommon event in all patients undergoing colonoscopy, occurring in 1 in every 4487 colonoscopy procedures. There are several explanations for ‘missing SPS’ by the health check-up centre endoscopist, gastroenterology department practice endoscopist or referring physicians (gastroenterologist), even by an expert gastroenterologist. SPS is rarely present; therefore, endoscopists may miss this condition. Nevertheless, endoscopists should be aware of the possibility of SPS and the definition or the 2010 WHO criteria. Gathering information from previous colonoscopy examinations, such as polyp numbers, polyp location, polyp size, and polyp histological findings of both the index lesion and all other lesions in the colon may be an important step in not missing SPS. Accurate recognition and assessment of SPS is the key to successful diagnosis. Regular education or training regarding current knowledge of SPS prevalence and criteria is important for endoscopists.

We found that the most common SPS criterion which patients in the general population met was “≥5 serrated polyps proximal to the sigmoid colon with two or more of these being >10 mm”. The 2010 WHO definitions for SPS states are less specific: it is necessary to specify in detail how many sequential colonoscopies were performed over time or the interval between the index and follow-up colonoscopy examinations. One pathologist suggested a personal approach in which polyps removed over time may be used to provide a cumulative total (15).

Some studies showed an association trend between SPS and extra-colonic malignancy (16). One study reported that 28% of SPS patients had extra-colonic cancer (17) and the other study reported that 24% had extra-colonic cancer (18), while Hazewinkel et al. (19) found 9 extra-colonic cancers, compared to 13 expected cancers in the general population among 105 SPS patients. Our data were either inconclusive or did not support this association. Accordingly, it was concluded that neither a reasonable association nor a causal relationship between SPS and extra-colonic malignancy could be established.

In conclusion, SPS patients were diagnosed late at screening colonoscopy. Our findings suggest those expert endoscopists (the health check-up centre or gastroenterology department practice) or referring physicians (gastroenterologist) with less awareness, pathological examination errors, failure to biopsy lesions adequately or follow-up errors may all be important factors in the failure to diagnose or detect SPS at colonoscopy. SPS may be associated with an increased risk of extra-colonic cancer; therefore, a colonoscopic procedure that fails to detect SPS may have life-threatening implications for the patient. Waiver of informed consent by Seoul National University Bundang Hospital IRB (IRB No: B-1512/328-116). The Seoul National University Bundang Hospital IRB waive the informed consent. This work is used in minimal risk research involving the retrospective medical records review.

## Figures and Tables

**Table 1:**
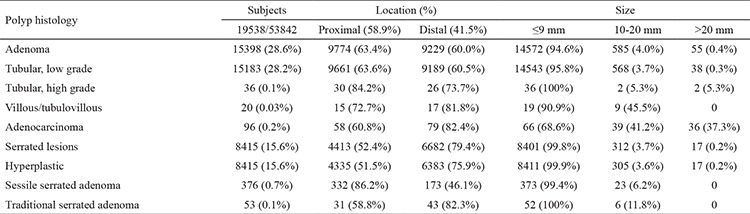
Baseline demographic data

**Table 2:**
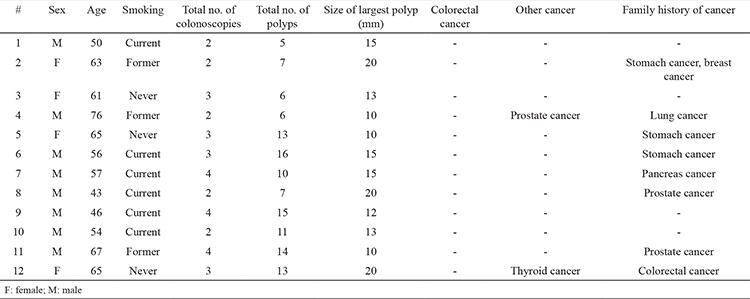
Characteristics of serrated polyposis syndrome patients
